# The Therapeutic Potential of Purmorphamine Across Disease Models

**DOI:** 10.1111/jcmm.71349

**Published:** 2026-09-04

**Authors:** Ashis Dhar, Ahnaf Dil Sharar, Saikarthik Papisetty, Mohammad Bydon, F. M. Moinuddin

**Affiliations:** ^1^ Department of Neurological Surgery University of Chicago Chicago Illinois USA; ^2^ University of Minnesota Medical School Minneapolis Minnesota USA

**Keywords:** Alzheimer, amyotrophic lateral sclerosis, autism, neurodegeneration, purmorphamine, small‐molecule, smoothen agonist, sonic hedgehog, stroke

## Abstract

Purmorphamine (PUR) is a trisubstituted purine compound that selectively activates Smoothened receptor, thereby initiating Sonic Hedgehog (Shh) signalling—a pathway critical for embryonic patterning, neuronal specification and tissue regeneration across multiple organ systems. Dysregulation of Shh signalling has been implicated in degenerative diseases yet therapeutic interventions targeting this pathway remain limited. PUR demonstrates broad therapeutic efficacy across diverse preclinical disease models by activating both canonical GLI‐mediated transcription and non‐canonical Shh pathways, resulting in neuroprotection, reduced neuroinflammation, enhanced blood–brain barrier integrity and tissue regeneration. In contrast to previous assumptions that Shh pathway activation requires endogenous ligand binding, PUR bypasses this requirement through direct Smoothened engagement, offering a pharmacologically tractable approach to pathway modulation. Preclinical studies demonstrate that PUR enhances motor neuron survival in amyotrophic lateral sclerosis models, protects dopaminergic neurons in Parkinson's disease, reverses behavioural abnormalities in autism spectrum disorder, promotes neurovascular repair following stroke, restores myelin integrity in multiple sclerosis models and drives osteogenic differentiation in bone tissue engineering applications. Beyond its established Smoothened agonist activity, recent evidence identifies PUR as a positive allosteric modulator of the secretin receptor, expanding its therapeutic scope to include cardiovascular applications such as hypertension management through enhanced nitric oxide bioavailability.

## 1 Introduction

A small molecule is a low molecular weight organic compound and is the most common type of compound used in drug development. Historically, examples of small molecule drugs include penicillin, aspirin and corticosteroids [1, 2]. Their advantages include simple structure, general stability, high bioavailability and the ability to be customized to meet specific therapeutic goals. Purmorphamine (PUR) is a small molecule characterized by its trisubstituted purine structure. PUR has been widely used as a tool compound to study and modulate Sonic Hedgehog (Shh) signalling both in vitro and in vivo models [3]. This application was first described by Wu et al. [4], who identified PUR as a small‐molecule agonist of Smoothened (Smo). It directly binds to the receptor Smo, functionally mimicking the endogenous Shh ligand and subsequently activating the Shh signalling pathway [5]. Shh plays a central role in coordinating the spatial and temporal dynamics of embryonic development, regulating pattern formation, cell fate specification and morphogenesis [6, 7]. Its importance extends beyond early development to tissue maturation and regenerative responses across multiple organ systems. Shh signalling has been implicated in the specification of neuronal subtypes, osteogenic differentiation, cardiovascular remodelling and maintenance of central nervous system architecture—highlighting its broad therapeutic potential in regenerative medicine [8].

Preclinical studies have demonstrated that PUR exerts beneficial effects in a variety of neurodegenerative and neurodevelopmental disease models, including stroke, amyotrophic lateral sclerosis (ALS) and autism spectrum disorder (ASD), by promoting neuroprotection, reducing neuroinflammation and enhancing tissue repair [9, 10, 11, 12, 13]. In addition, PUR demonstrates potent neuroprotective effects by protecting hippocampal neurons under oxidative stress, exhibiting both protective and regenerative properties. Interestingly, despite its Shh‐activating properties, PUR has also been reported to exert anti‐hypertensive effects [14].

**FIGURE 1:**
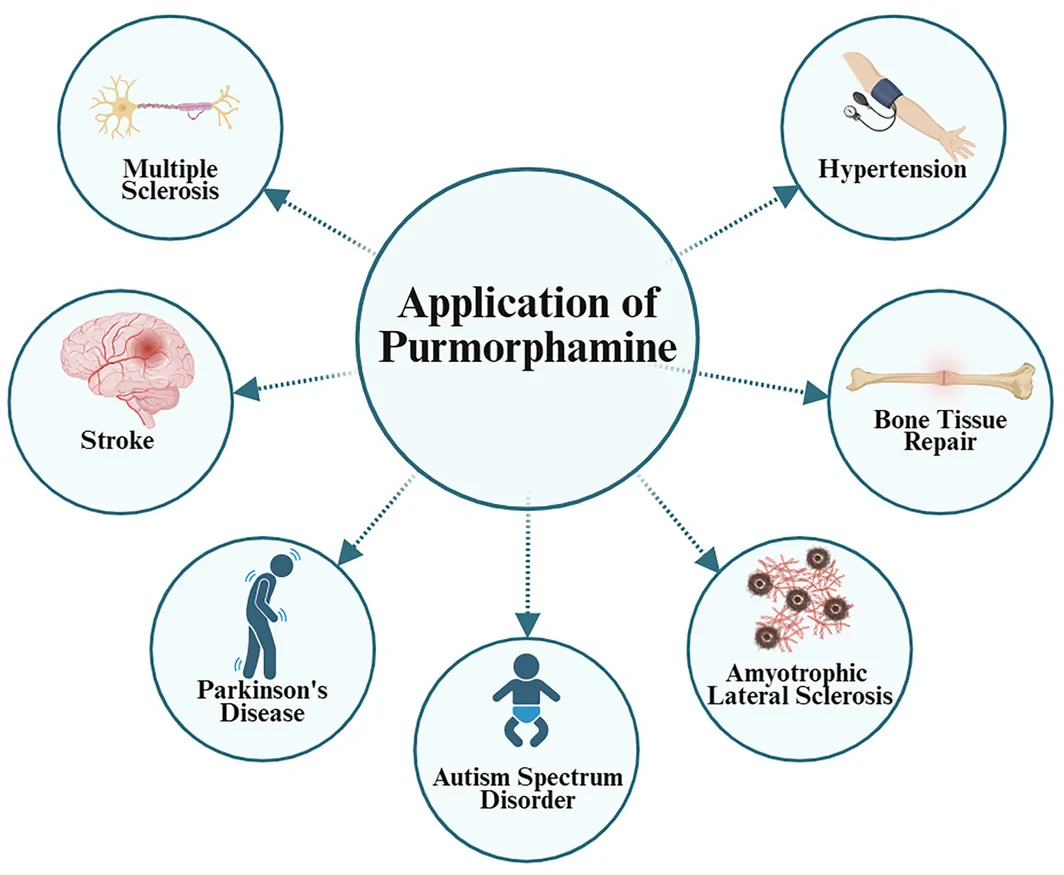
Application of purmorphamine.

As a small molecule, PUR represents a unique candidate for regenerative therapy due to its ability to cross biological barriers and engage both canonical and non‐canonical Shh signalling mechanisms [5, 15, 16]. In this review, we discuss the role of PUR in various diseases and explore its future potential in regenerative drug. (Figure 1).

## 2 Beyond General SMO Agonism: The Pharmacological Case for PUR

Pharmacological modulation of SMO has been approached through structurally diverse compounds, including SAG, GSA‐10, oxysterol derivatives and PUR, and prior reviews have comprehensively surveyed this broader landscape [17, 18, 19]. However, PUR occupies a distinct position within this class that justifies dedicated synthesis rather than a comparative survey, for three reasons. First, unlike SAG or GSA‐10, which function exclusively through SMO, PUR has been identified as a positive allosteric modulator of SCTR, demonstrating antihypertensive activity lasting over 50 h after a single dose and confirmed oral bioavailability properties no other SMO agonist currently possesses [14]. Second, while prior reviews covered the broader Shh modulator field through 2014, the intervening decade has produced a substantial body of PUR‐specific preclinical literature spanning neurodegeneration, neuropsychiatric disease, demyelinating disorders, neural precursor biology and metabolic regulation that has not been critically synthesized [9, 11, 20, 21]. Third, a critical knowledge gap persists: it remains unclear whether PUR's reported neurological effects represent consistent compound‐specific actions or primarily reflect the general consequences of SMO‐Shh activation findings that span neuronal survival, glial responses, neuroinflammation, oxidative injury and tissue repair but remain dispersed across disease‐specific models, leaving their mechanistic consistency and translational relevance unresolved. This review addresses that gap by identifying reproducible mechanisms across models, distinguishing PUR‐associated effects from broader SMO agonism, and critically evaluating pathway specificity, dosing and comparative efficacy building the evidentiary framework needed to advance PUR from an experimental tool compound toward a viable therapeutic candidate. No prior review has systematically evaluated PUR across these disease contexts; a gap this review directly addresses. (Table 1).

**TABLE 1:** Purmorphamine in the context of representative pharmacological modulators of SMO/SHH signalling.

Modulator	Modulatory action	General characteristics/Relevance	Clinical status	Key references
Purmorphamine (PUR)	SMO agonist	Synthetic trisubstituted purine; activates Shh signalling without requiring endogenous ligand; crosses the blood–brain barrier; oral bioavailability demonstrated; also identified as a positive allosteric modulator of the secretin receptor	Preclinical only; no clinical trials registered	[4, 5, 14]
SAG (Smoothened Agonist)	SMO agonist	Synthetic chlorobenzothiophene‐derived agonist; widely used experimental tool for Shh pathway activation; structurally distinct from PUR; well‐characterized in CNS and developmental models	Preclinical only	[17]
GSA‐10 and related agonists	SMO/Hedgehog pathway agonism	Represents additional synthetic approaches to Hedgehog pathway activation; distinct chemical scaffolds from PUR and SAG; studied primarily in developmental and stem cell contexts	Preclinical only	[19]
Oxysterol‐derived agonists	Positive modulation of SMO/Shh signalling	Endogenous lipid‐derived molecules (e.g., 20(S)‐OHC) capable of promoting Hedgehog pathway activity; bind to a distinct site on SMO from cyclopamine and PUR	Preclinical only	[18]
Cyclopamine and related antagonists	SMO antagonism	Classical steroidal alkaloid inhibitor of SMO; instrumental in defining small‐molecule regulation of the Shh receptor; parent compound of the vismodegib pharmacophore	Preclinical tool compound; parent scaffold evolved into clinical candidates	[17, 22]
Vismodegib (GDC‐0449)	SMO antagonist	First FDA‐approved SMO inhibitor (2012); approved for advanced basal cell carcinoma; orally bioavailable; demonstrates that SMO is a clinically validated pharmacological target	FDA‐approved (basal cell carcinoma, 2012)	[23]
Sonidegib (LDE225)	SMO antagonist	Second FDA‐approved SMO inhibitor (2015); approved for locally advanced basal cell carcinoma; further validates SMO as a druggable oncology target	FDA‐approved (basal cell carcinoma, 2015)	[24]

Abbreviations: CNS, central nervous system; FDA, U.S. Food and Drug Administration; PUR, purmorphamine; SAG, smoothened agonist; SAH, subarachnoid haemorrhage; SCTR, secretin receptor; SHH, Sonic Hedgehog; SMO, Smoothened.

### 2.1 Mechanism and Translational Implications

PUR is a tri‐substituted purine small molecule and a Smo agonist. Its IUPAC name is 9‐cyclohexyl‐N‐(4‐morpholinophenyl)‐2‐(naphthalen‐1‐yloxy)‐9H‐purin‐6‐amine [3] The compound has the molecular formula C_31_H_32_N_6_O_2_ and a molecular weight of 520.62 g/mol. Structurally, PUR's purine core distinguishes it from the chlorobenzothiophene‐derived Hh‐Ag1.x/SAG class of SMO agonists. PUR is commercially available and their chemical properties are described in Table 2 [25].

**TABLE 2:** Chemical properties of purmorphamine (PUR) [25].

Purmorphamine (PUR)	
Chemical structure	[25] 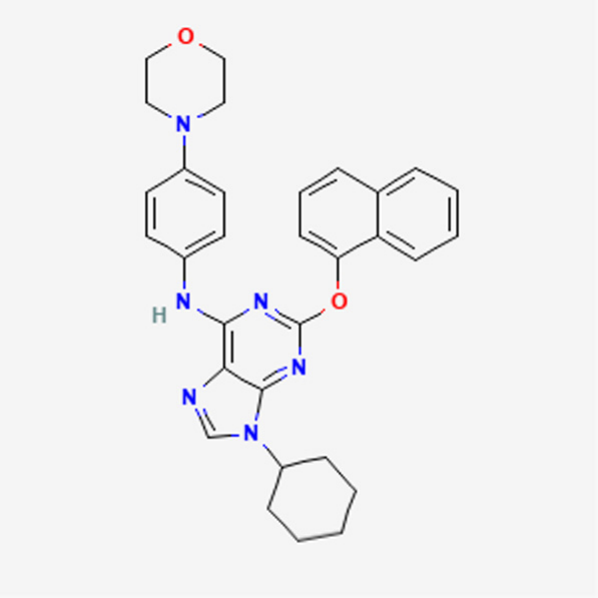
Molecular weight	520.62
Formula	C_31_H_32_N_6_O_2_
Storage	Store at −20°C
Purity	≥ 98% (HPLC)
Solubility	DMSO to 100 mM
Appearance	Solid
Colour	White to off‐white
Potency	Micromolar potency; EC₅₀ ~0.5–1 μM in Hedgehog activation assays
Binding site	Direct agonist at the cyclopamine‐binding pocket of Smo

PUR offers a unique mechanism for selectively activating the Shh pathway without requiring endogenous ligand binding. It mimics canonical Shh signalling by relieving Patched1 (Ptch1)‐mediated inhibition of Smo. PUR bypasses the need for ligand interaction and directly binds to Smo, initiating downstream intracellular signalling cascades [3, 26, 27, 28].

PUR activates both canonical (GLI1‐mediated) and non‐canonical Shh signalling pathways by targeting Smo [29]. In the canonical pathway, PUR‐mediated Smo activation leads to the nuclear translocation of GLI transcription factors, particularly GLI1, which upregulates Shh target genes. This includes the activation of intracellular Ca^2+^ signalling, PI3K/Akt and ERK/MAPK pathways, as well as cytoskeletal remodelling [27].

In addition to canonical signalling, PUR also engages non‐canonical Shh pathways that function independently of GLI‐mediated transcription. These Smo‐dependent, non‐canonical mechanisms regulate intracellular calcium flux, actin cytoskeletal remodelling and microtubule dynamics [15, 30, 31].

PUR confers neuroprotection through a combination of GLI1‐driven transcriptional activity and rapid non‐transcriptional mechanisms, such as ERK phosphorylation and intracellular Ca^2+^ buffering. Importantly, PUR modulates astrocyte function by inducing proliferation, elongation [32, 33] and GFAP upregulation via GLI‐ and Akt‐dependent pathways. These changes enhance astrocytic roles in glutamate clearance, potassium buffering and trophic support, particularly under injury conditions [33, 34, 35].

Beyond astrocytes, PUR exerts anti‐inflammatory effects on microglia. It suppresses pro‐inflammatory cytokines like TNF‐α and IL‐1β [27] while promoting anti‐inflammatory mediators primarily through Akt pathway activation [20]. This signalling shift drives microglia toward a reparative, M2‐like phenotype and reduces neuronal damage caused by microglial activation.

**FIGURE 2:**
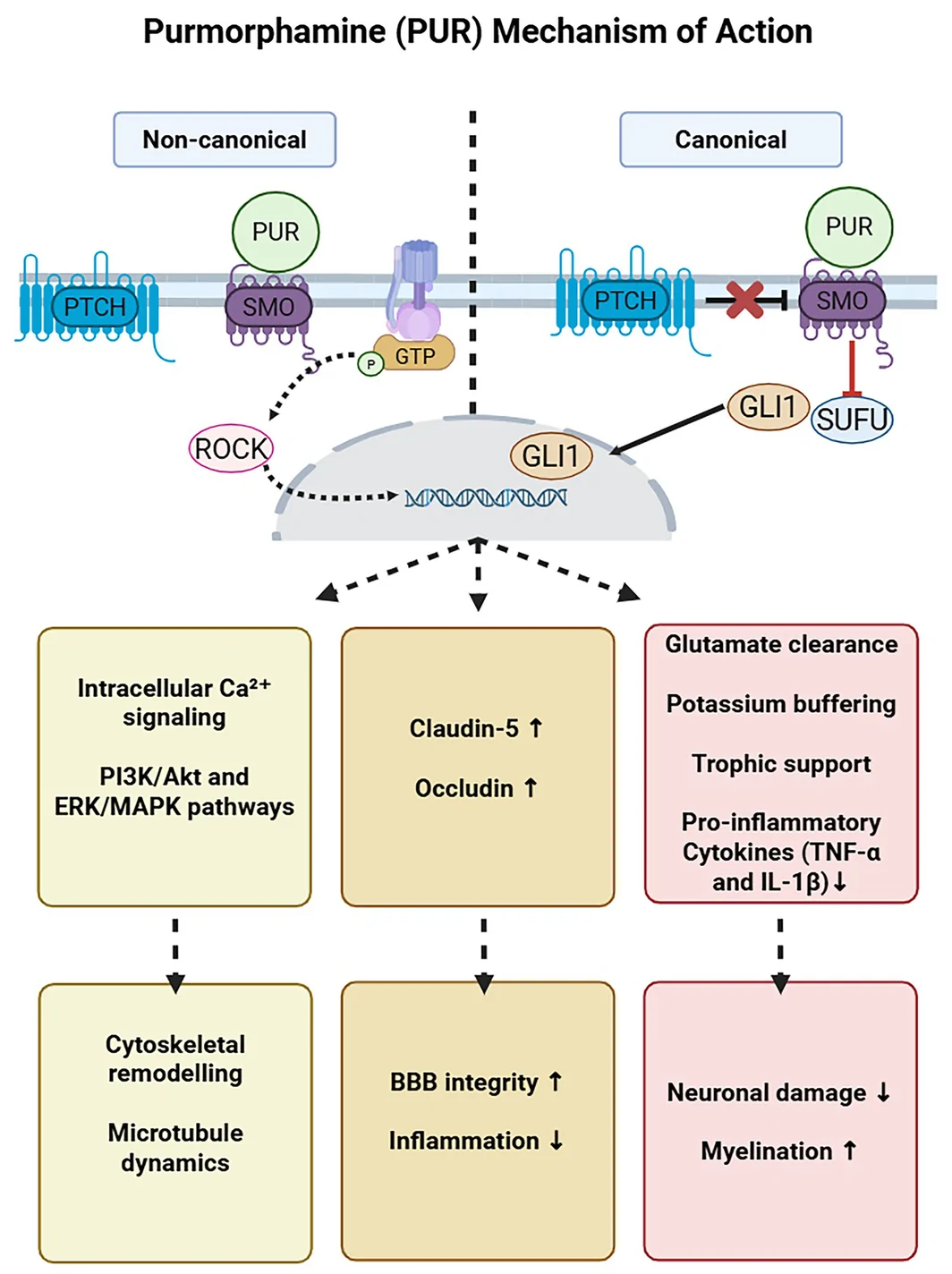
Overview of purmorphamine (PUR) mechanism of action.

Lastly, PUR supports blood–brain barrier (BBB) integrity [9]. Smo activation enhances the expression of tight junction proteins, including claudin‐5 and occludin, thereby reducing paracellular permeability in inflammatory or ischemic contexts [5, 36, 37, 38]. (Figure 2, Table 3).

**TABLE 3:** Purmorphamine (PUR): Therapeutic applications, experimental models and expanding research landscape.

Disease/Application	Year	Species/Model	Dose & regimen	Route	Key findings	Ref.
Ischemic stroke	2020	Rat	5 mg/kg for 2 days	i.p.	↑ Neuronal viability, neuroligin, neurexin; ↓ NF‐κB signalling	[39]
Ischemic stroke	2014	Mouse	1, 5, & 15 mg/kg single dose	i.v.	↓ Inflammation, astrogliosis, BBB permeability; ↑ neurogenesis & neurovascularization	[40]
ALS	2022	Mouse, SOD1G93A	15 mg/kg every 3 days (from 9 weeks)	i.p.	↑ Survival & motor performance; PI3K/Akt involvement	[20]
ALS	2025	Mouse, hSOD1	15 mg/kg every 3 days	i.p.	↑ FAK/ERK activity; delayed disease progression; improved muscle strength	[35]
Parkinson's disease	2017	Mouse, MPTP‐induced	1 mg/kg, 1 h pre‐MPTP	i.p.	↓ Neuroinflammation; ↑ motor function; protected SNpc DA neurons; PI3K/Akt‐dependent	[27]
Autism spectrum disorder	2021	Rat, propionic acid model	5 & 10 mg/kg for 32 days	i.p.	Behavioural & biochemical rescue; ↑ MBP; ↓ oxidative damage; restored Bax/Bcl‐2	[12]
Obsessive‐compulsive disorder	2022	Rat, 8‐OHDPAT intraDRN model	5 & 10 mg/kg daily (Days 12–44)	i.p.	↓ Compulsive behaviours; synergy with fluvoxamine	[9]
Multiple sclerosis	2024	Rat, EB‐ICP induced MS	5 & 10 mg/kg	i.p.	↑ Locomotion, cognitive & spatial memory; ↓ TNF‐α, IL‐1β, NEFL; enhanced remyelination	[11]
Blood–brain barrier disruption	2014	MBEC4 endothelial monolayer ± astrocyte CM	0.01–1 μM for 24 h	In vitro	↑ ZO‐1, occludin, claudin‐5; ↓ IL‐1β‐induced leak	[37]
Hypertension	2022	Spontaneously hypertensive rats	30 & 50 mg/kg	i.v. & oral	↓ Blood pressure ~14%; effects up to 50 h; oral bioavailability confirmed; SCTR positive allosteric modulator	[14]
Osteogenic differentiation	2005	Human BMMSCs, C3H10T1/2 (in vitro)	1–3 μM	In vitro	↑ ALP, Runx2, mineralization; osteoblast lineage commitment enhanced	[41]
Neural precursor‐cell injury	2024	Wistar rat (in vivo); NE‐4C cells (in vitro)	~1 μM (in vitro); not specified (in vivo)	In vitro/i.p.	Restored Shh signalling suppressed by nonylphenol; ↑ NPC proliferation in SGZ; rescued Sox2+ and Ki67+ cells	[42]
Alcohol withdrawal	2024	Rat (voluntary drinking, 16–20 sessions)	5 mg/kg prior to each alcohol session	i.p.	↓ Anxiety‐like behaviour post‐withdrawal; altered Hh cascade expression in brain; modulated Smo/Gli signalling	[43]
Obesity/Metabolic disease	2025	Mouse (in vivo)	Not specified	Systemic	Potentiates SCTR–cAMP–PKA signalling; promotes brown adipose thermogenesis; non‐classical SMO‐independent pharmacology	[21]
Neuronal stress/Neuroplasticity	2026	N2a cells (mouse neuroblastoma, in vitro)	1 μM co‐treated with dexamethasone for 24 h	In vitro	Restored GR phosphorylation, Shh components, BDNF & profilin1; partial rescue of neurite architecture & proliferation	[44]
Oligodendrocyte biology/Myelination	2026	In vitro & in vivo (mixed)	Variable (context‐dependent)	In vitro/i.p.	Excessive SMO activation inhibits OL differentiation & myelination; dose‐ and cell‐type‐dependent effects established	[45]

Abbreviations: ALS, amyotrophic lateral sclerosis; ASD, autism spectrum disorder; BBB, blood–brain barrier; BMMSCs, bone marrow mesenchymal stem cells; DA, dopaminergic; EB‐ICP, ethidium bromide‐intracerebral peduncle; GR, glucocorticoid receptor; Hh, Hedgehog; i.p., intraperitoneal; i.v., intravenous; MBP, myelin basic protein; MPTP, 1‐methyl‐4‐phenyl‐1,2,3,6‐tetrahydropyridine; MS, multiple sclerosis; NEFL, neurofilament light chain; NPC, neural precursor cell; OL, oligodendrocyte; SCTR, secretin receptor; SGZ, subgranular zone; SMO, Smoothened; SNpc, substantia nigra pars compacta; SOD1, superoxide dismutase 1.

### 2.2 PUR in Bone Tissue Repair

Bone‐related pathologies such as major fractures and maxillofacial defects demand more than the intrinsic reparative properties of bone; they require robust regenerative stimulation mediated by osteogenesis [46, 47]. Mesenchymal stem cells (MSCs), as multipotent progenitors, have been demonstrated to differentiate into osteoblasts, chondrocytes, adipocytes, myocytes and other cell types essential for tissue regeneration [48, 49]. Their fate is tightly regulated by signalling pathways including Shh, which influences osteoblast lineage processes [50]. PUR acts as a potent artificial modulator of Shh signalling and has been shown to drive osteoblastic differentiation in multipotent MSCs [41]. One of the most consistent and early effects of PUR‐induced osteogenic differentiation is the enhancement of alkaline phosphatase (ALP) activity, a classical marker of early‐stage osteoblast maturation [41, 51]. ALP plays a central role not only as an indicator of lineage processes but also as a functional contributor to bone matrix mineralization by hydrolyzing pyrophosphate, an inhibitor of hydroxyapatite formation [52, 53]. In mesenchymal progenitor models such as C3H10T1/2 and rat bone marrow–derived MSCs, PUR treatment significantly increases ALP activity in a dose‐ and time‐dependent manner, often surpassing conventional osteoinductive agents like BMP‐2 and dexamethasone in both intensity and temporal onset [3, 54, 55, 56]. This enhancement reflects the activation of the Shh pathway, wherein PUR directly stimulates Smo, leading to GLI1/GLI2‐mediated transcription of osteogenic regulators such as RUNX2 [3], which subsequently drives ALPL gene expression. Experimental data consistently demonstrate that PUR‐treated cells exhibit earlier and more robust ALP staining and calcium deposition, indicating that PUR accelerates osteoblast lineage commitment and promotes extracellular matrix maturation [3, 41].

In addition to enhancing ALP, PUR upregulates transcription factors such as Cbfa1/Runx2, which are master regulators of osteoblast commitment [57, 58]. It also induces the expression of hedgehog pathway mediators (SMO, PTCH1, GLI1, GLI2), further amplifying downstream gene transcription, including RUNX2 and BMPs, both of which are crucial for osteogenic lineage progression [59]. In vitro studies employing C3H10T1/2 cells, a mesenchymal‐like cell line capable of multi‐lineage differentiation, have established PUR's ability to stimulate osteoblastic pathways. Enhanced ALP activity and bone‐like nodule formation were noted upon PUR treatment [4]. Importantly, PUR demonstrated synergistic effects with BMP‐4, promoting trans‐differentiation of preadipocytes and myoblasts into osteoblasts [60]. This synergy enhances the therapeutic potential of PUR in orthopaedic and dental applications, including osseointegration of implants.

A 2011 in vitro study using Light II cells further extended this framework by combining PUR with tricalcium phosphate (β‐TCP) as a bone adhesive, which led to enhanced osteoblastic activity and suggested new bioengineering applications [5, 61, 62]. Mechanistically, PUR activates Smo, which then stimulates GLI transcription by acting on its promoter region, initiating transcriptional cascades essential for osteogenesis [63].

### 2.3 Therapeutic Implication of PUR in ALS

ALS is a devastating, progressive neurodegenerative disorder marked by the selective degeneration of motor neurons in the spinal cord and motor cortex. Motor neuron loss manifests clinically as progressive muscle atrophy, respiratory failure and a median survival of 2–5 years post‐diagnosis [64]. Despite advances in supportive care, there is currently no cure. In ALS, the Shh signalling pathway is suppressed by cerebrospinal fluid–borne inhibitors that suppress Gli activity despite adequate or elevated Shh ligand levels. Multiple cell‐based ALS models have demonstrated persistent suppression of downstream Shh signalling, suggesting a blockade distal to ligand‐receptor interaction. In particular, studies using mutant SOD1 (mSOD), expressing motor neuron‐like cells, have shown that Gli‐responsive luciferase reporter assays yield significantly reduced transcriptional output even in the presence of Shh, indicating impaired signalling beyond the Patched‐Smo axis [65]. Peterson et al. [66] showed that PUR restores cell viability and reduces apoptotic markers in mSOD‐expressing cells subjected to oxidative stress. These observations raise the possibility that enhancing Gli transcriptional activity beyond PUR‐mediated Smo activation could offer additional therapeutic benefit in ALS.

In hSOD1 mice, ALS pathogenesis is associated with altered expression of Shh, focal adhesion Kinase (FAK) and extracellular signal regulated kinase (ERK) proteins. Interestingly, administration of PUR to 90‐day‐old hSOD1 mice enhanced FAK/ERK pathway activity, thereby delaying disease progression and improving muscle strength [35]. These findings suggest that PUR could be a potential therapeutic agent targeting this novel pathway for motor neuron diseases.

In parallel, Shh is essential for maintaining BBB integrity via astrocyte‐derived Shh signalling to endothelial cells, which promotes tight junction protein expression (ZO‐1, Claudin‐5) and suppresses inflammatory mediators such as IL 1β and ICAM 1, thereby maintaining immune quiescence in the CNS vasculature [37, 67]. Thus, PUR may confer indirect but potent protection to the neurovascular unit via astrocytic Shh stimulation, offering a multidimensional therapeutic effect that extends beyond neuron‐intrinsic survival.

### 2.4 Impact of PUR on Neurodevelopmental Outcomes in ASD

ASD encompasses a range of neurodevelopmental disturbances characterized by impairments in cognitive flexibility, social interaction and repetitive behaviours. Mounting evidence implicates dysregulation of the Shh–Smo signalling axis in the pathogenesis of ASD, particularly during early neurodevelopment. This pathway plays a critical role in cortical lamination, hippocampal morphogenesis and axonal tract organization. Disruption of Shh signalling has been associated with increased oxidative stress, abnormal neurogenesis and compromised BBB integrity [10, 40].

One environmental factor known to perturb this pathway is propionic acid (PPA), a short‐chain fatty acid produced by gut microbiota [68]. Elevated PPA levels have been reported in the faeces of children with ASD, suggesting a link between gut dysbiosis and neurodevelopmental pathology [69, 70]. Rodent models utilizing intracerebroventricular PPA administration reproduce core ASD‐like phenotypes, including repetitive behaviours, impaired social interaction, neuroinflammation and demyelination [71]. At the molecular level, PPA affects Smo activation, impairs oligodendrocyte maturation and activates intrinsic apoptotic pathways via elevated caspase‐3 activity and a dysregulated Bax/Bcl‐2 ratio [13, 72].

Consistent with its action in other neurodegenerative and developmental contexts, Rahi et al. [12] demonstrated that PUR treatment reverses behavioural and molecular abnormalities induced by PPA in rats, primarily through reactivation of the Shh pathway and downstream anti‐inflammatory and myelination‐related effects. Notably, PUR enhances expression of myelin basic protein (MBP), attenuates oxidative damage (e.g., reduced malondialdehyde levels) and rebalances apoptotic signalling via normalization of Bax and Bcl‐2 expression [10, 40, 73].

PUR, through Smo‐mediated stimulation of the Shh‐Gli axis, upregulates Olig2 and Nkx2.2 expression, promoting oligodendrocyte precursor specification and re‐engaging developmental myelination that is often dysregulated in ASD [74, 75, 76]. Prajapati et al. [11] demonstrated using a rat model that Olig2‐expressing progenitors contribute not only to oligodendrogenesis but also to glial homeostasis under neuroinflammatory stress, positioning PUR as a pathway‐corrective agent capable of restoring myelin integrity through precise transcriptional reprogramming.

### 2.5 Role of PUR in Parkinson's Disease

Parkinson's disease (PD) affects approximately 1%–3% of individuals over the age of 60, with prevalence projected to rise by over 30% by 2030 due to demographic aging and extended life expectancy [77, 78]. Pathologically, PD is marked by the progressive degeneration of dopaminergic neurons in the substantia nigra pars compacta (SNpc), along with loss of noradrenergic neurons in the locus coeruleus, resulting in motor dysfunction characterized by resting tremor, bradykinesia and rigidity [79].

Recent insights have identified the Shh signalling pathway as a critical modulator of dopaminergic neuronal survival [80]. Activation of the Shh–Smo axis induces downstream signalling through the PI3K/Akt pathway, thereby suppressing oxidative stress, inflammation and apoptosis in neuronal populations [27]. These insights have led to therapeutic exploration of small‐molecule Smo agonists such as PUR, which functionally substitute for Shh ligands to reactivate canonical signalling in vulnerable dopaminergic networks.

In a study by Shao et al. [27], PUR treatment in MPTP‐induced mouse models of PD resulted in increased levels of Gli1 and phosphorylated Akt, indicating robust activation of the Shh and PI3K/Akt signalling cascades. Additionally, PUR‐treated mice exhibited improved outcomes in traction, pole and rotarod tests, indicative of enhanced motor coordination and preservation of nigrostriatal pathway integrity. Notably, PUR suppresses microglial activation via SHH–PI3K/Akt signalling, as evidenced by reduced Iba1 expression, while inhibition with LY294002 (a PI3K inhibitor) [81] reverses this effect. Moreover, as also shown in this article, Shao et al. [27] demonstrated that SHH activation downregulates pro‐inflammatory cytokines (IL‐1β, TNF‐α) and upregulates the anti‐inflammatory cytokine TGF‐β1, supporting earlier findings that microglial activation precedes dopaminergic degeneration in early PD pathogenesis [82]. These findings indicate that PUR treatment maintains the structural and functional integrity of dopaminergic circuits, offering a neuroprotective advantage beyond mere cell survival.

Beyond its neuroprotective properties, PUR may also hold therapeutic value in alleviating levodopa‐induced dyskinesia (LID), a common and debilitating side effect of prolonged dopamine replacement therapy. In a 6‐hydroxydopamine (6‐OHDA) mouse model of PD subjected to chronic L‐DOPA treatment, Malave et al. [83] demonstrated that PUR co‐administration significantly reduced abnormal involuntary movements (AIMs) in a dose‐dependent manner. This behavioural improvement was mechanistically linked to the modulation of cholinergic interneurons (CINs), which are known contributors to LID pathophysiology. CINs express Smo and are responsive to Shh signalling; in dyskinetic conditions, they exhibit hyperactivity and disrupted firing patterns. PUR treatment restored Shh‐dependent signalling within CINs, leading to a rebalancing of their excitability toward physiological levels, thereby mitigating the hypercholinergic drive underlying dyskinesia.

### 2.6 PUR as a Neuroprotective Agent in Stroke

Ischemic stroke is one of the most common neurological diseases causing tissue degeneration, patient disabilities or death. Shh was found to be upregulated in neurons during ischemia, and inhibition of the Shh pathway aggravated ischemic damage in rats. PUR, as an agonist of the Shh pathway, appears a potent neuroprotective agent after ischemic stroke.

Chechneva et al. [40] reported that PUR administration significantly increased expression of doublecortin (DCX), a marker of neuroblast migration, and GAP‐43, a key protein involved in neurite outgrowth and axonal remodelling in a mouse model of cerebral ischemia. These changes were accompanied by upregulation of synaptophysin, indicating enhanced synaptic density and plasticity in the peri‐infarct cortex. Moreover, the presence of BrdU^+^/CD31^+^ co‐labelled cells pointed to endothelial proliferation and angiogenic remodelling. Together, these findings suggest that PUR promotes coordinated neurovascular repair after stroke by enhancing both neural and vascular regeneration, supporting its potential as a pharmacological strategy to extend the reparative effects of endogenous Shh signalling.

Beyond its effects on neurovascular remodelling and oxidative stress, PUR also modulates key molecular regulators of neuronal survival and synaptic integrity. In ischaemic models, PUR treatment reduces activation of cleaved caspase‐3 and lowers the Bax/Bcl‐2 ratio—hallmarks of the intrinsic (mitochondrial) apoptotic pathway [10].

Furthermore, PUR promotes phosphorylation of Akt and CREB, two key effectors downstream of PI3K and Shh signalling. Akt activation inhibits pro‐apoptotic pathways and supports metabolic resilience, while CREB activation drives the transcription of neurotrophic and anti‐apoptotic genes necessary for repair and plasticity. These findings collectively highlight PUR's ability to orchestrate a multi‐axis neuroprotective response—combining anti‐apoptotic signalling, mitigation of oxidative stress, synaptic maintenance and BBB stabilization [22, 39].

Hu et al. using a preclinical subarachnoid haemorrhage (SAH) model, reported that intraperitoneal PUR restored phosphorylation of extracellular signal‐regulated kinase 1/2 (ERK1/2), reduced the Bcl‐2‐associated X protein/B‐cell lymphoma 2 (Bax/Bcl‐2) ratio and upregulated nuclear factor erythroid 2–related factor 2 (Nrf2) and heme oxygenase‐1 (HO‐1) expression in the prefrontal cortex. These effects were abolished by co‐treatment with the Smo antagonist Cyclopamine, confirming a Smo‐dependent mechanism. Behavioural outcomes further supported PUR's neuroprotective efficacy [22].

### 2.7 Role of PUR in Experimental Models of Multiple Sclerosis

Multiple sclerosis (MS) is a complex and unresolved neurological disorder characterized by impaired oligodendrocyte survival, demyelination and disruption of axonal connectivity. These pathological changes lead to a broad spectrum of clinical manifestations, including visual impairment, dizziness, numbness, weakness, urinary urgency, motor dysfunction, fatigue, cognitive deficits, depression and ultimately, death [84, 85].

Prajapati et al. [11] modelled MS pathology via intracerebral peduncle injection of ethidium bromide (EB) in rats [11, 86], and assessed disease progression through biological and behavioural evaluations. Their study investigated the combined therapeutic effects of Fingolimod, the first FDA‐approved orally administered drug for MS, and PUR [87, 88, 89, 90]. The combination of PUR and Fingolimod preserved brain‐to‐body weight ratio and enhanced neurological outcomes in EB‐induced MS rats. Within 26 days, treated animals showed superior cognitive retention, spatial memory and locomotor recovery compared to Fingolimod alone. PUR (10 mg/kg) more effectively restored Smo–Shh protein levels, induced a stronger anti‐apoptotic Bax/Bcl profile and improved glutamate clearance, acetylcholine restoration and monoamine (dopamine, serotonin) preservation. Combination therapy also produced greater reductions in TNF‐α and IL‐1β, alongside marked attenuation of oxidative stress. While these findings are promising, they would benefit from further validation. Specifically, confirming the mechanistic link between Smo activation and Shh/Gli signalling would strengthen the results.

### 2.8 Repurposing PUR for Hypertension via Secretin Receptor Modulation

Despite being a well‐known Smo agonist, Kailash et al. identified PUR as a novel modulator of the secretin receptor (SCTR) for treating hypertension. Secretin (SCT), traditionally characterized as a gastrointestinal peptide hormone, also participates in fluid homeostasis and vascular regulation. In vivo studies demonstrate that secretin deficiency leads to elevated blood pressure, whereas exogenous administration lowers systemic arterial pressure by enhancing endothelial nitric oxide synthase (eNOS) activity and promoting nitric oxide (NO) bioavailability [14]. PUR eliminates current treatment drawbacks: short half‐life, high costs and lack of an oral drug. Researchers used docking‐based virtual screening and identified over 14 million compounds then finalized 21 based on binding affinity. In vitro and ex vivo tests confirmed PUR acts as a modulator of SCT for SCTR activation as a positive allosteric modulator that amplifies SCT's effects by 204%. Additionally, from derivatives, the morpholine core was identified as necessary for allosteric binding to SCTR. Furthermore, in hypertensive rats, PUR reduced blood pressure by around 14% with effects lasting up to 50 h after intravenous administration. Importantly, the compound showed oral bioavailability and a much longer half‐life compared to STC.

## 3 Conclusions

Although PUR demonstrates promising therapeutic potential across multiple disease models, it lacks the regulatory precision of endogenous transcription factors, which can selectively bind DNA and orchestrate gene expression with high fidelity. Still, PUR effectively modulates Shh signalling to promote neuroprotection, vascular stability and anti‐inflammatory responses.

To advance PUR toward clinical application, future work must develop delivery methods that restrict activation to disease‐relevant regions and cell types. Approaches such as receptor‐targeted nanoparticles, biodegradable scaffolds or locally administered formulations could reduce systemic exposure and enhance selectivity. Similar strategies have been proposed to mitigate off‐target risks in other Shh‐based therapies, particularly given the pathway's known association with fibrosis and tumorigenesis.

## Author Contributions

**Ashis Dhar:** conceptualization, writing – review and editing, writing – original draft, data curation. **Ahnaf Dil Sharar:** writing – review and editing. **Saikarthik Papisetty:** writing – review and editing. **Mohammad Bydon:** supervision. **F. M. Moinuddin:** conceptualization, writing – review and editing, writing – original draft, supervision.

## Funding

The authors have nothing to report.

## Conflicts of Interest

The authors declare no conflicts of interest.

## Data Availability

Data sharing not applicable to this article as no datasets were generated or analysed during the current study.
